# Endometrial microbiota in women with and without adenomyosis: A pilot study

**DOI:** 10.3389/fmicb.2023.1075900

**Published:** 2023-01-20

**Authors:** Qi Lin, Hua Duan, Sha Wang, Zhengchen Guo, Sirui Wang, Yanan Chang, Chao Chen, Minghong Shen, Hejun Shou, Chang Zhou

**Affiliations:** ^1^Department of Minimally Invasive Gynecology, Beijing Obstetrics and Gynecology Hospital, Capital Medical University, Beijing Maternal and Child Health Care Hospital, Beijing, China; ^2^Department of Gynecology, Shengli Clinical Medical College of Fujian Medical University, Fujian Provincial Hospital, Fuzhou, China

**Keywords:** adenomyosis, endometrium, endometrial microbiota, microbiota diversity, microbiota function

## Abstract

**Introduction:**

The endometrial microbiota plays an essential role in the health of the female reproductive system. However, the interactions between the microbes in the endometrium and their effects on adenomyosis remain obscure.

**Materials and methods:**

We profile endometrial samples from 38 women with (*n*=21) or without (*n*=17) adenomyosis to characterize the composition of the microbial community and its potential function in adenomyosis using 5R 16S rRNA gene sequencing.

**Results:**

The microbiota profiles of patients with adenomyosis were different from the control group without adenomyosis. Furthermore, analysis identified *Lactobacillus zeae, Burkholderia cepacia, Weissella confusa, Prevotella copri,* and *Citrobacter freundii* as potential biomarkers for adenomyosis. In addition, *Citrobacter freundii, Prevotella copri*, and *Burkholderia cepacia* had the most significant diagnostic value for adenomyosis. PICRUSt results identified 30 differentially regulated pathways between the two groups of patients. In particular, we found that protein export, glycolysis/gluconeogenesis, alanine, aspartate, and glutamate metabolism were upregulated in adenomyosis. Our results clarify the relationship between the endometrial microbiota and adenomyosis.

**Discussion:**

The endometrial microbiota of adenomyosis exhibits a unique structure and *Citrobacter freundii, Prevotella copri*, and *Burkholderia cepacia* were identified as potential pathogenic microorganisms associated with adenomyosis. Our findings suggest that changes in the endometrial microbiota of patients with adenomyosis are of potential value for determining the occurrence, progression, early of diagnosis, and treatment oadenomyosis.

## 1. Introduction

Adenomyosis (ADS) is one of the most challenging problems in benign uterine disease. This is because the mechanisms associated with this condition remain unclear and there are no unified and effective treatment options at present. The Endometriosis Committee of the Chinese Medical Doctor Association of Obstetricians and Gynecologists defines ADS as an eutopic endometrium that invades and migrates into the uterine myometrium, a condition that affects 7–23% of women of reproductive age (García-Solares et al., 2018; Leng et al., 2020). The recognized mechanisms of ADS include the invasion of the endometrial basalis into the myometrium, microtrauma of the endometrial-myometrial interface, genetics, immune disorders, and inflammatory mediators (An et al., 2017; Vannuccini et al., 2017; Guo, 2020; Zhai et al., 2020; Guo et al., 2022). However, the clinical presentations of patients with ADS are heterogeneous, thus indicating that unknown pathological mechanisms may exist (Bourdon et al., 2021).

Several studies have proposed that ADS is associated with an imbalance in the vagina microbiota (Chen et al., 2020; Chao et al., 2021; Kunaseth et al., 2022). *Lactobacillus* is known to dominate the bacterial composition of a healthy vagina and deters the growth of opportunistic pathogens; however, dysbiosis can potentially lead to the occurrence of disease. Chronic pelvic pain (CPP) has been associated with both endometriosis (EM) and ADS. The vaginal microbiota of patients with EM/ADS-associated CPP is known to feature a higher abundance of *Clostridium butyricum*, *Clostridium disporicum*, *Alloscardovia omnicolens,* and *Veillonella montpellierensis* (Chao et al., 2021). In a previous study, Kunaseth et al. (2022) reported that *Alloscardovia*, *Oscillospirales*, *Ruminoccoccaceae*, *Oscillospiraceae*, *Enhydrobacter*, *Megamonas*, *Selenomonadaceae,* and *Faecalibacterium* all showed an increased abundance in the vaginal microbiota of patients with ADS. *Atopobium* is a known biomarker for patients with EM combined with ADS of the vaginal microbiota (Chen et al., 2020).

The uterus has long considered as a sterile environment. However, several studies have shown that the endometrium is colonized by bacterial strains that differ from the vaginal microbiota (Moreno et al., 2016; Chen et al., 2017). Dysbiosis of the endometrial microbiota is likely to lead to endometrial cancer (Walther-António et al., 2016; Sobstyl et al., 2022), polyps (Fang et al., 2016), endometriosis (Akiyama et al., 2019; Wessels et al., 2021), endometritis (Moreno et al., 2018; Hernandes et al., 2020), and recurrent pregnancy loss (Łaniewski et al., 2020). A recent study confirmed that *Porphyromonas somerae* represents a suitable biomarker for endometrial cancer (Walsh et al., 2019). Fang et al. (2016) found that *Lactobacillus*, *Bifidobacterium*, *Gardnerella,* and *Streptococcus* were increased in women with endometrial polyps. A previous study found that the relative abundances of the *Streptococcaceae*, *Moraxellaceae*, *Staphylococcaceae*, and *Enterobacteriaceae* families were significantly increased among females with endometriosis (Akiyama et al., 2019). However, research on the endometrial microbiota in patients with ADS is rare. In this study, we aimed to investigate the composition and biomarkers of the microbiota in endometrial samples from females with and without ADS using 5R 16S rRNA gene sequencing (Nejman et al., 2020; Poore et al., 2020). We also investigated whether biomarkers of the endometrial microbiota could be used to diagnose ADS and then predict the potential interactions between microbiota and ADS.

## 2. Materials and methods

The study was performed according to the guidelines of the Declaration of Helsinki and was approved by the Ethics Committee of the Beijing Obstetrics and Gynecology Hospital, Capital Medical University No. 2016-KY-012-02.

### 2.1. Patients

**Figure 1:**
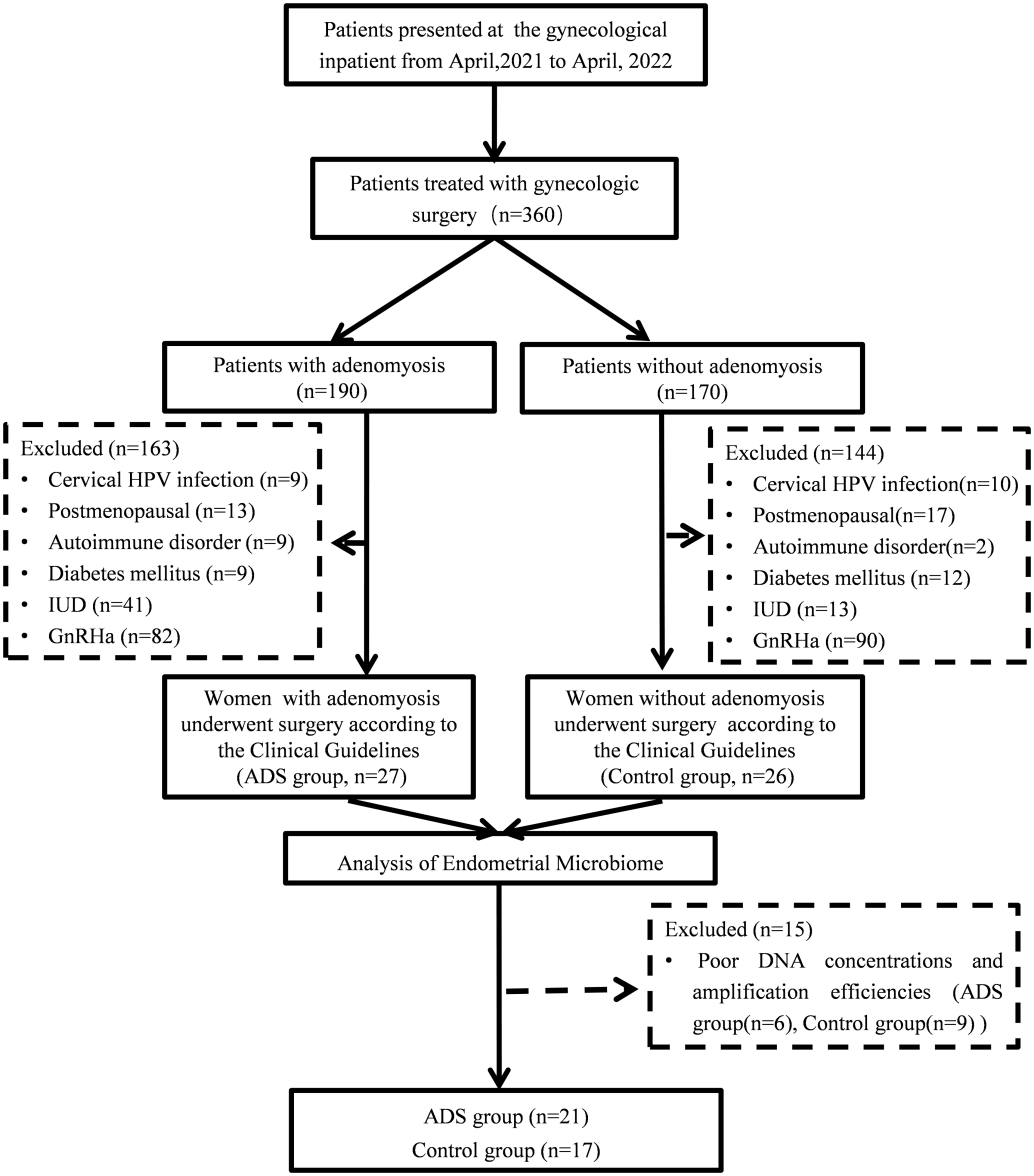
Flow chart showing patient enrollment.

In total, we recruited 360 females who received laparoscopic hysterectomy and were pathologically diagnosed with and without adenomyosis between April 2021 and April 2022 at the Institute of Minimally Invasive Gynecology, Beijing Obstetrics and Gynecology Hospital, Capital Medical University. All participants were fully informed and provided signed informed consent in accordance with the Declaration of Helsinki. The diagnosis of adenomyosis was confirmed by histological examination by two pathologists as the presence of ectopic endometrial tissue (endometrial stroma and glands) within the myometrium. The exclusion criteria were as follows: (1) postmenopausal women; (2) women who received preoperative hormone or gonadotropin-releasing hormone agonist (GnRHa) therapy for at least 3 months prior to sampling; (3) women who were diagnosed with endometrial disease, such as endometrial carcinoma and atypical endometrial hyperplasia; (4) women who had been confirmed with acute genital tract inflammation or with diseases of the lower urinary tract; (5) women with chronic diseases, including autoimmune disorders, diabetes mellitus, coronary heart disease, and hypertension; (6) women with cervical HPV infection and fever; (7) women with intrauterine devices, and (8) patients who had taken antibiotics over the last 3 months before sample collection (except mandatory prophylactic antibiotic treatment before surgery). Three hundred and seven patients (163 from the ADS group and 144 from the control group) were excluded from this study. Besides, 15 endometrial samples (6 from the ADS group and 9 from the control group) did not present PCR products for sequencing and were considered negative. Thirty-eight participants (twenty-one from the ADS group and seventeen from the control group) were ultimately included in the final analysis. The overall procedure is outlined in Figure 1.

### 2.2. Endometrial samples

Samples were collected from participants during surgery. Following hysterectomy, the ‘Y’ shape uterus was immediately opened with a sterile knife by incising along the central line from the fundus to the external uterine ostium and then exposing each uterine horn. Specimens for subsequent research were obtained from the fundus uteri with a sterile cotton swab, including the posterior and anterior wall of the endometrium. Each specimen was placed in a sterile tube and immediately transported to the laboratory in an icebox. Samples were subsequently stored in a −80°C refrigerator to await DNA extraction.

### 2.3. DNA extraction, 5R 16S rRNA gene amplification, and sequencing

Genomic DNA was extracted from samples by CTAB methods and 1% agarose gels were used to determine the concentration and quality of DNA. The five (V2, V3, V5, V6, and V8) regions on the 16S rDNA were amplified with specific primers (F1, 5′-TGGCGAACGGGTGAGTAA-3′; F2, 5′-ACTCCTACGGGAGGCAGC-3′; F3, 5′-GTGTAGCGGTGRAATGCG-3′; F4, 5′-GGAGCATGTGGWTTAATTCGA-3′; F5, 5′-GGAGGAAGGTGGGGATGAC-3′; R1, 5′-AGACGTGTGCTCTTCCGAAGACGTGTGCTCTTCCGA-3′; R2, 5′-AGACGTGTGCTCTTCCGATCTGTATTACCGCGGCTGCTG-3′; R3, 5′-AGACGTGTGCTCTTCCGATCTCCCGTCAATTCMTTTGAGTT-3′; R4, 5′-AGACGTGTGCTCTTCCGATCTCGTTGCGGGACTTAACCC-3′; R5,5′-AGACGTGTGCTCTTCCGATCTAAGGCCCGGGAACGTATT-3′; FF1,5′-AATGATACGGCGACCACCGAGATCTACACTCTTTCCCTACACGACGCTCTTCCGATCTTGGCGAACGGGTGAGTAA-3′; FF2, 5′-AATGATACGGCGACCACCGAGATCTACACTCTTTCCCTACACGACGCTCTTCCGATCTACTCCTACGGGAGGCAGC-3′; FF3, 5′-AATGATACGGCGACCACCGAGATCTACACTCTTTCCCTACACGACGCTCTTCCGATCTGTGTAGCGGTGRAATGCG-3′; FF4, 5′-AATGATACGGCGACCACCGAGATCTACACTCTTTCCCTACACGACGCTCTTCCGATCTGGAGCATGTGGWTTAATTCGA-3′; FF5, 5′-AATGATACGGCGACCACCGAGATCTACACTCTTTCCCTACACGACGCTCTTCCGATCTGGAGGAAGGTGGGGATGAC-3′; RR5, 5′-CAAGCAGAAGACGGCATACGAGAT-NNNNNNNN-GTGACTGGAGTTCAGACGTGTGCTCTTCCGATCT-3′). Polymerase chain reaction (PCR) was carried out with Phusion Hot Start Flex 2X Master Mix (New England Biolabs, M0536L). The cycling conditions for PCR were as follows: 98°C for 30 s, followed by 30 cycles of 98°C for 10 s, 62°C for 10 s, 62°C for 15 s, and another 5 min at 72°C. Subsequently, PCR products were purified by applying the E.Z.N.A. Gel Extraction Kit and libraries were sequenced on an Illumina NovaSeq 6,000 system (LC-Bio, Hangzhou, China). Sampling controls (blank control), DNA extraction controls (negative control), and no-template PCR amplification controls (negative control) were also utilized. The Short Multiple Regions Framework (SMURF) method was used to read counts from the five regions into coherent profiling results, classify bacterial taxonomy, and analyze the relative abundance of bacteria (Fuks et al., 2018; Nejman et al., 2020). Sequence reads were normalized to reduce the impact of low abundance noise when the raw reads were less than 1,000 and the relative abundance of bacteria was less than 10^−4^. Moreover, we used the prevalence of bacteria in the negative control samples to determine the abundance of contaminating bacteria from sampling and experimental processes. The threshold for identifying contaminated bacteria was set at a prevalence of 50%. The GreenGenes database (version: 2013^1^) was used as a reference database for the SMURF method.

### 2.4. Statistical analysis

In this study, we used a blinded approach that the analyst unaware of the patients group allocation in all statistical analyses. Clinical statistical analysis was performed in SPSS software (version 25.0). Continuous variables were analyzed by t-tests while categorical variables were analyzed by Fisher’s exact test; *p <* 0.05 was considered statistically significant. The raw sequence reads were analyzed by the vsearch and usearch tools in Rstudio. A bacterial abundance table was generated to analyze the differences in bacterial profiles between the ADS and control group. R and STAMP v.2.1.3. were used to visualize the taxonomic composition of microbial communities. Alpha diversity was computed using the Mann–Whitney test and *p* < 0.05 was considered statistically. Rarefaction curves were used to estimate whether the sample size could comprehensively reflect diversity. Community clustering was measured by principal component analysis (PCA) and analyzed by the Adonis test; *p* < 0.05 was considered statistically significant. The Linear Discriminative Analysis Effect Size (LEfSe) method was used to identify distinct endometrial microbiota between different groups and explore potential biomarkers (Segata et al., 2011; Parks et al., 2014).^2^ Phylogenetic Investigation of Communities by Reconstruction of Unobserved States (PICRUSt, version 2.0^3^) (Douglas et al., 2019) was performed to identify the different functions of the endometrial bacteria and to profile Kyoto Encyclopedia of Genes and Genomes (KEGG) Orthology functions.^4^ Statistically significant results (*p* < 0.05) were presented in the figures by STAMP v2.1.3 (Parks et al., 2014).

## 3. Results

### 3.1. Sociodemographic and samples

A total of 53 participants were included in this study and each participant provided endometrial samples; 21 samples were obtained from ADS patients and 17 samples obtained from patients without adenomyosis (the control group). These samples were used for PCR and produced high qualify DNA. All the recruited participants received hysterectomy and their surgical and pathological reports were available for analysis. The mean age was 45.82 ± 5.090 years in the control group and 45.71 ± 4.649 years in the ADS group; there was no significant difference between the two groups in terms of age (*p* = 0.9453). Furthermore, no significant differences were found in terms of body mass index (BMI), menstrual cycle phase, parity, or contraceptive method when compared between the two groups (*p* = 0.4406, *p* = 0.5768, *p* = 0.4474, and *p* = 0.7029, respectively). Table 1 shows the clinical features of the patients at baseline.

**Table 1:** Clinical characteristics of the participants.

Characteristics	Control (*n* = 17)	Adenomyosis (*n* = 21)	*p*-value
Age, years (mean ± SD)	45.82 ± 5.090	45.71 ± 4.649	0.9453
BMI, kg/m^2^	21.69 ± 0.8281	21.48 ± 0.7867	0.4406
Menstrual cycle phase n/N (%)			0.5768
Follicular	15/17 (88.24)	20/21 (95.24)	
Luteal	2/17 (11.76)	1/21 (4.76)	
Parity n/N (%)			0.4474
Nullipara	1/17 (5.88)	0/21 (0)	
Multipara	16/17 (94.12)	21 (100)	
Contraception, n/N (%)			0.7029
Nil	5/17 (29.41)	4/21 (28.57)	
Condoms	12/17 (70.59)	17/21 (71.43)	

### 3.2. Sequencing coverage

5R 16S rRNA gene analysis was performed on the remaining 21 patients from the ADS group and 17 from the control group. A total of 3,703,825 raw reads (97,469.07895 reads per sample) and 3,277,880 clean reads (86,260 reads per sample) were obtained from 38 samples with an average Q30 percentage of 91.12% (Supplementary Table S1). The alpha rarefaction curve indicated a reasonable sequencing depth and Good’s coverage was almost 0.99 in the two groups (Supplementary Figure S1).

### 3.3. Identification of endometrial microbiota

**Figure 2:**
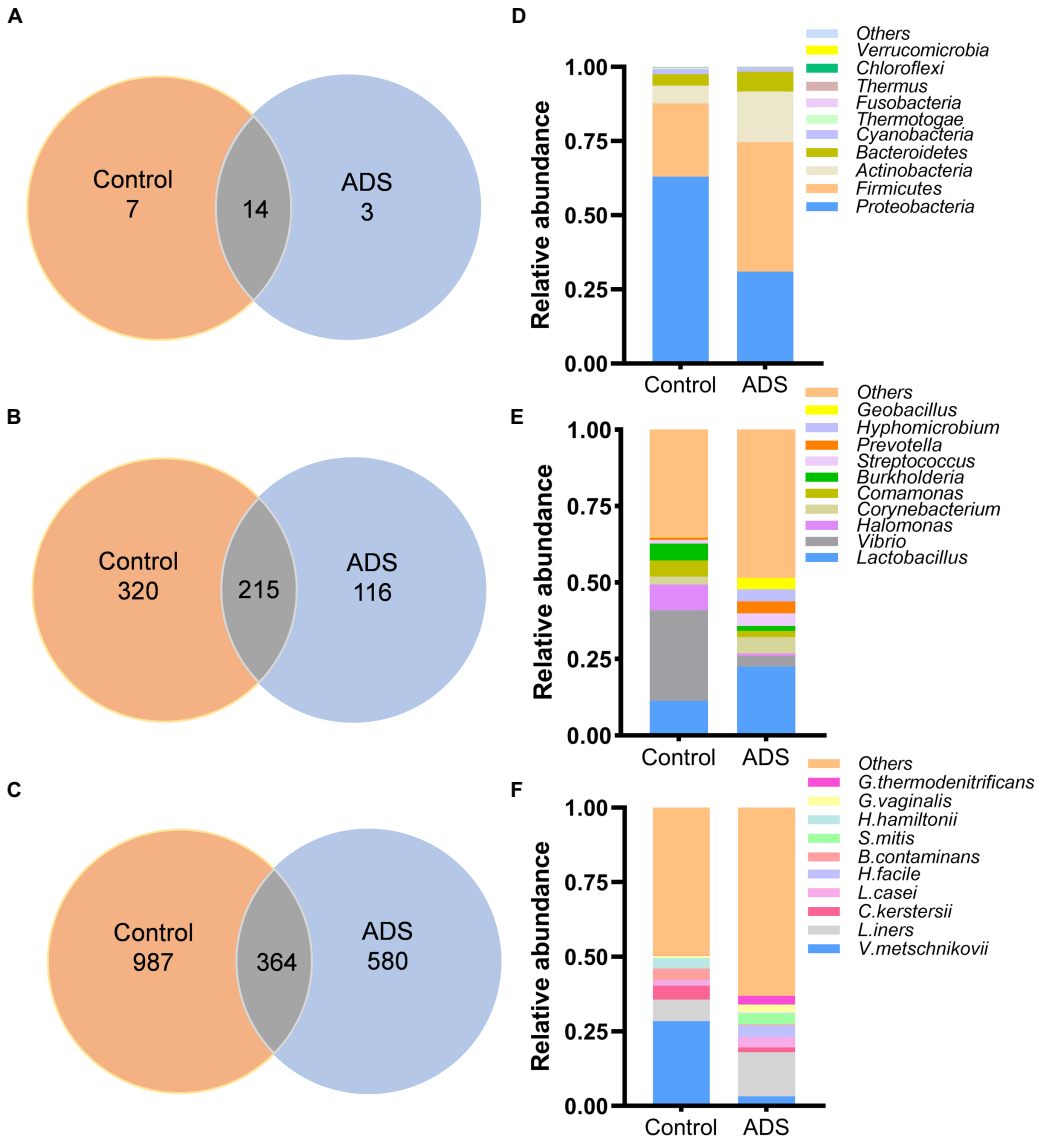
Disparities in bacterial distribution between the adenomyosis (ADS) group and control group. Venn diagram representing the total number of bacterial at the phylum **(A)**, genus **(B)**, and species **(C)** levels; Colum chart showing the top 10 taxa of endometrial microbiota between the two groups at the phylum **(D)**, genus **(E)**, and species **(F)** levels. *V. metschnikovii, Vibrio metschnikovii*; *L. iners, Lactobacillus iners*; *C. kerstersii, Comamonas kerstersii*; *B. contaminans, Burkholderia contaminans*; *H. hamiltonii, Halomonas hamiltonii*; *L. casei, Lactobacillus casei*; *G. vaginalis, Gardnerella vaginalis*; *G. thermodenitrificans, Geobacillus thermodenitrificans*; *H. facile, Hyphomicrobium facile*; *S. mitis, Streptococcus mitis*.

The distribution of endometrial microbiota differed between the two groups at the phylum, genus, and species levels. A total of 24 phyla, 651 genera, and 1,931 species were detected (Figures 2A–C). The distribution of the top 10 taxa of the endometrial microbiota at the phylum, genus, and species levels is depicted in Figures 2D–F. At the phylum level (Figure 2D), the bacterial distribution in the ADS group was dominated by *Firmicutes* (43.61%), followed by *Proteobacteria* (31%), *Actinobacteria* (17.11%), and *Bacteroidetes* (6.68%). However, most species in the control group were *Proteobacteria* (62.94%), followed by *Firmicutes* (24.68%), *Actinobacteria* (6.09%), *Bacteroidetes* (3.86%), and *Cyanobacteria* (1.66%). At the genus level (Figure 2E), *Lactobacillus* (22.58%), *Corynebacterium* (5.32%), *Streptococcus* (4.17%), *Prevotella* (3.94%), *Hyphomicrobium* (3.90%), *Geobacillus* (3.78%), and *Vibrio* (3.34%) were the predominant abundant bacterial in the ADS group. In contrast, *Vibrio* (29.71%) were predominant in the control group. *Lactobacillus iners* (*L. iners*), belonging to the *Firmicutes* phylum, was the most dominant bacteria in the ADS group at the species level, while the main proportion in the control group was *Vibrio metschnikovii* (*V. metschnikovii*; phylum: *Proteobacteria*) (Figure 2F).

### 3.4. Diversity analysis of the endometrial microbiota between the two groups: Resource identification initiative

**Figure 3:**
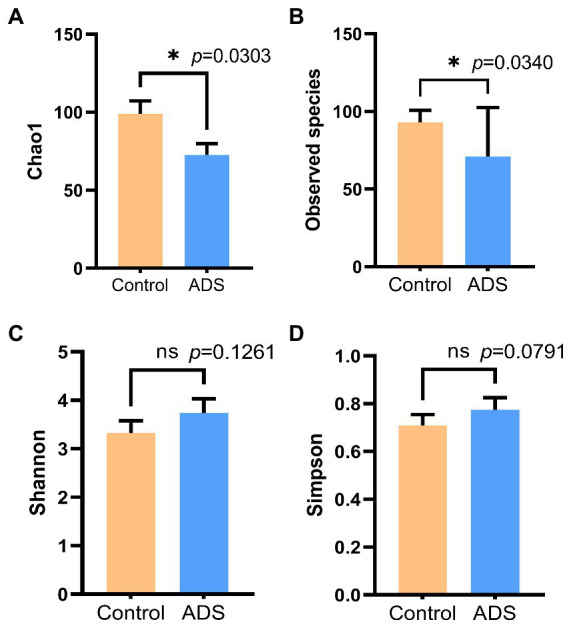
Alpha diversity analysis of the endometrial microbiota of women with or without adenomyosis. Chao1 **(A)**, Observed species **(B)**, Shannon index **(C)**, and Simpson index **(D)** were analyzed by the Mann–Whitney test. *p < 0.05 was statistically significant.

To estimate and determine the endometrial microbiota richness and diversity, we performed alpha and beta diversity analysis. Alpha diversity, including Chao1, Shannon index, Simpson index, and Observed species, was used to generate four column diagrams. As shown in Figure 3, there was no difference in the Shannon and Simpson indices when compared between the two groups (*p* > 0.05). In contrast, the Chao1 (*p* = 0.0303) and Observed species (*p* = 0.0340) in the control group were significantly higher than in the ADS group (*p* < 0.05). These results demonstrated a significant reduction in the endometrial microbial richness in patients with adenomyosis.

**Figure 4:**
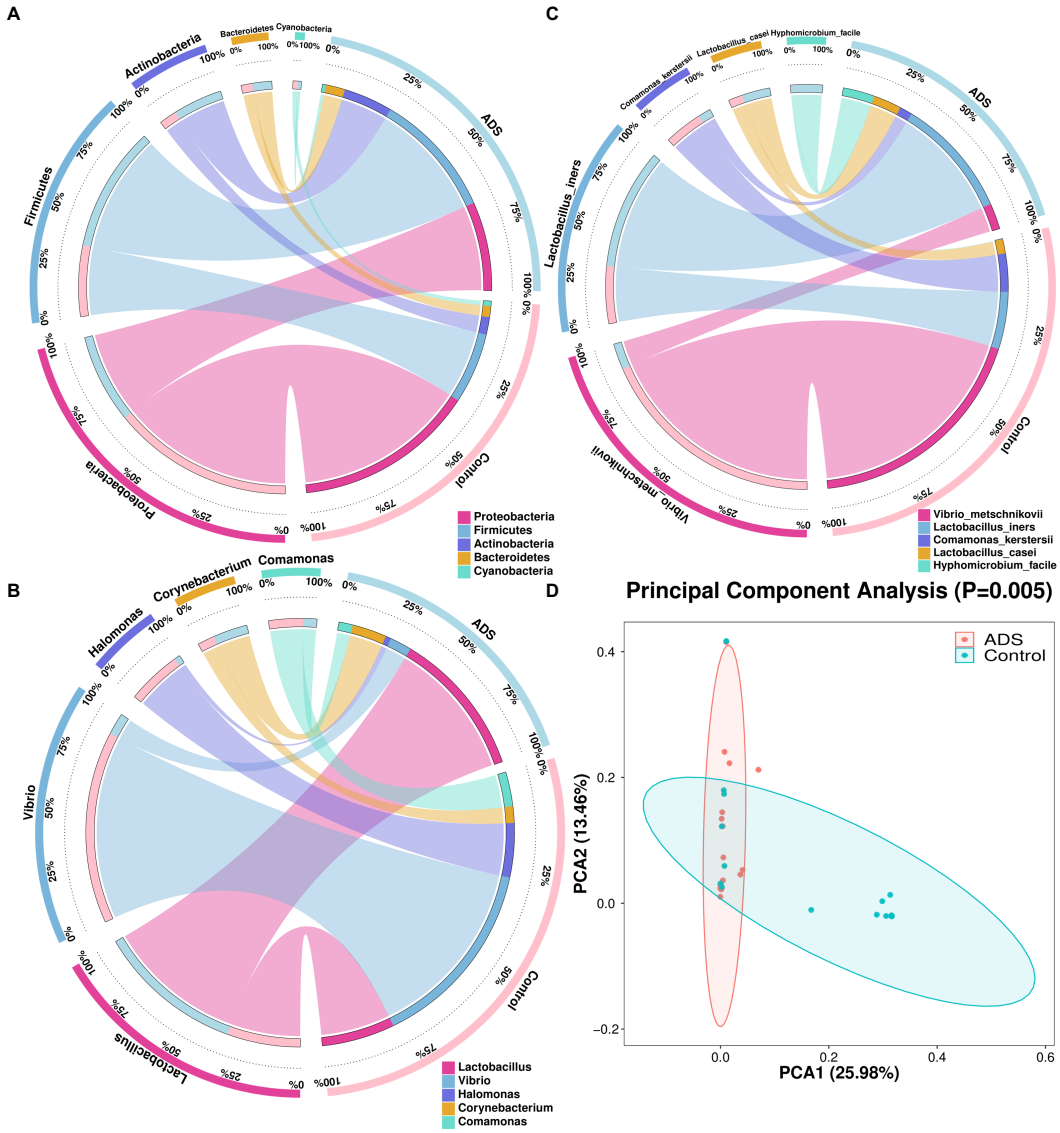
Endometrial microbiota were clustered according to adenomyosis or control grouping. Five different bacteria are presented in this figure with the relative abundance of the top five (TOP 5) at the phylum **(A)**, genus **(B),** and species **(C)** levels. The two groups and the relative proportions of each bacterium are shown in the right half of the circle. The five bacteria and the proportions occupied by each group are shown in the left half part of the circle. Principal Components Analysis (PCA) plots were also conducted **(D)**. Red dots indicate the adenomyosis (ADS) group while blue dots indicate the control group. Ovals represent the 95% CI for each group. The percentage on the *X* (PCA1) and *Y* (PCA2) axis explains the degree of each variation. Adonis tests were performed to calculate the statistical significance.

In addition, we also performed beta diversity analysis to reveal differences in the endometrial microbiota structure between the two groups. As previously shown in Figure 2, for patients with adenomyosis, *Firmicutes* and *Actinobacteria* were the most significant bacteria at the phylum level when compared with the control group. *Lactobacillus* was more dominant than the control group at the genus level. At the species level, *Lactobacillus iners* (*L. iners*), *Halomonas hamiltonii* (*H. hamiltonii*), and *Streptococcus mitis (S. mitis)* were more abundant than in the control group. The relative abundance of the five top-ranked bacteria in the endometrial microbiota was selected for further analysis among the two groups. In total, five differential bacterial phyla, genera, and species were depicted in a circular diagram (Figures 4A–C). These results are consistent with the previous conclusion that *Lactobacillus* and *Corynebacterium* dominated in adenomyosis. In contrast, *Vibrio*, *Halomonas,* and *Comamonas* were dominant in the control group. Principal component analysis (PCA) was used to analyze the relative abundance of bacteria and beta diversity index was determined by Bray–Curtis analysis at the species level. Figure 4D shows that the distribution and structure of the endometrial flora in the ADS group were significantly separate and different from the control group (*p* = 0.005).

### 3.5. Analysis of biomarkers

**Figure 5:**
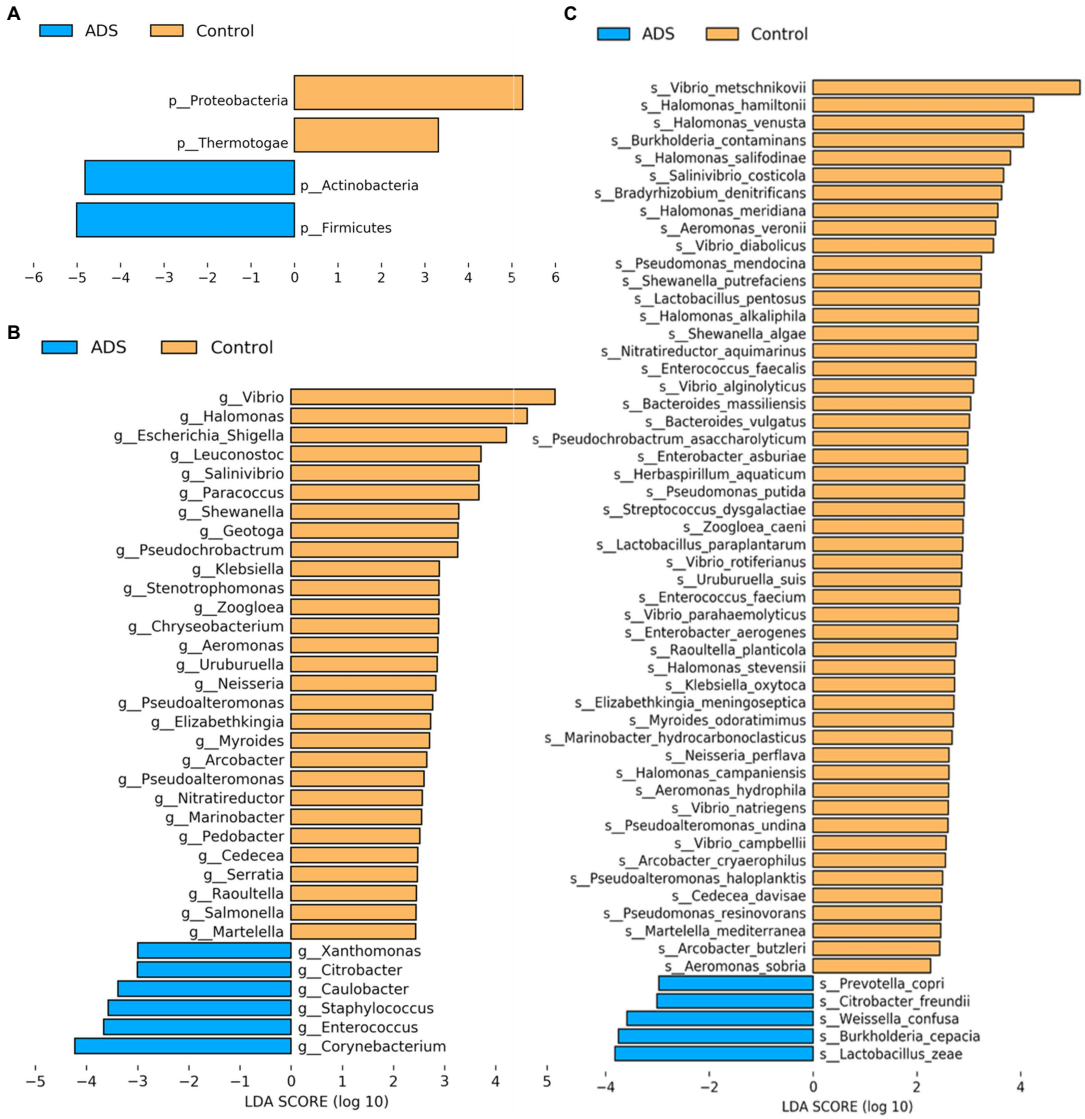
Analysis of putative biomarkers for adenomyosis. LEfSe analysis revealed the enrichment of taxa, including bacteria in the *Firmicutes* and *Actinobacteria* phylum, *Corynebacterium*, *Enterococcus*, *Staphylococcus*, *Caulobacter*, and *Citrobacter* genus, and *Lactobacillus zeae*, *Burkholderia cepacia*, *Weissella confusa*, *Citrobacter freundii*, *and Prevotella copri* species in patients with adenomyosis. The control group showed enrichment of the *Proteobacteria* and *Thermotogae* phylum, *Vibrio*, *Halomonas*, *Escherichia, Shigella*, *Leuconostoc*, *Salinivibrio*, *Paracoccus*, *Shewanella*, *Geotoga*, and *Pseudochrobactrum* genus. Histogram of LDA scores (log10) showing bacteria that presented with higher relative abundance in the ADS group (blue) when compared to the control group (orange) at the phylum **(A)**, genus **(B)**, and species **(C)** levels. Only statistically significant differences (*p* < 0.05) are shown.

**Figure 6:**
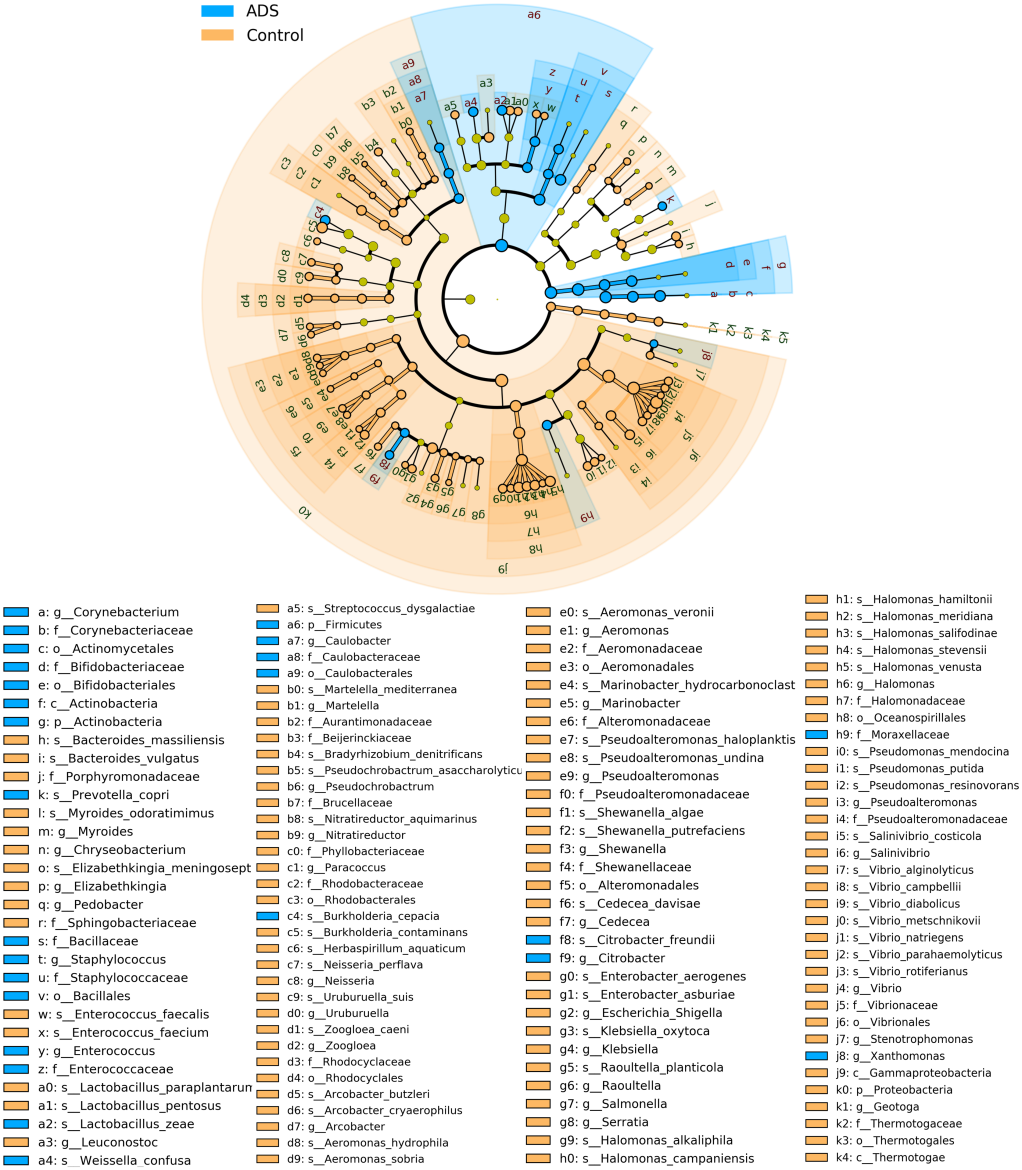
Cladogram report. The circle radiating from the inside to outside represents the classification level (from phylum to genus or species). Species without significant differences are colored yellow. Biomarkers were shown by specific colors in each group; the blue nodes indicate the bacteria that play an important role in the adenomyosis (ADS) group while the orange nodes indicate the microbial groups that play an important role in the control group. Prefixes represent abbreviations for each taxon: phylum (p_), class (c_), order (o_), family (f_), genus (g_), species(s_). Only statistically significant differences (*p* < 0.05) are shown.

Next, we attempted to identify differences in the composition of microbiota between groups by using the linear discriminant analysis (LDA) effect size (LEfSe) algorithm to discover potential biomarkers between the two groups (Figure 5). Using an LDA > 2.0 as a threshold for discriminative features, our analysis identified significant differences in the abundance of *Firmicutes* and *Actinobacteria* at the phylum level; these were more abundant in the ADS group than in the control group (Figure 5A). In addition, we found that *Corynebacterium*, *Enterococcus*, *Staphylococcus*, *Caulobacter,* and *Citrobacter* were significantly more abundant in the ADS group when compared with the control group at the genus level (Figure 5B). Furthermore, *Lactobacillus zeae* (*L. zeae*), *Burkholderia cepacia* (*B. cepacia*), *Weissella confusa* (*W. confusa*), *Citrobacter freundii* (*C. freundii*), and *Prevotella copri* (*P. copri*) were significantly more abundant in the ADS group at the species level (Figure 5C). Identical results were generated by cladogram analysis (Figure 6).

### 3.6. Analysis of biomarkers for the prediction and diagnosis of adenomyosis

**Figure 7:**
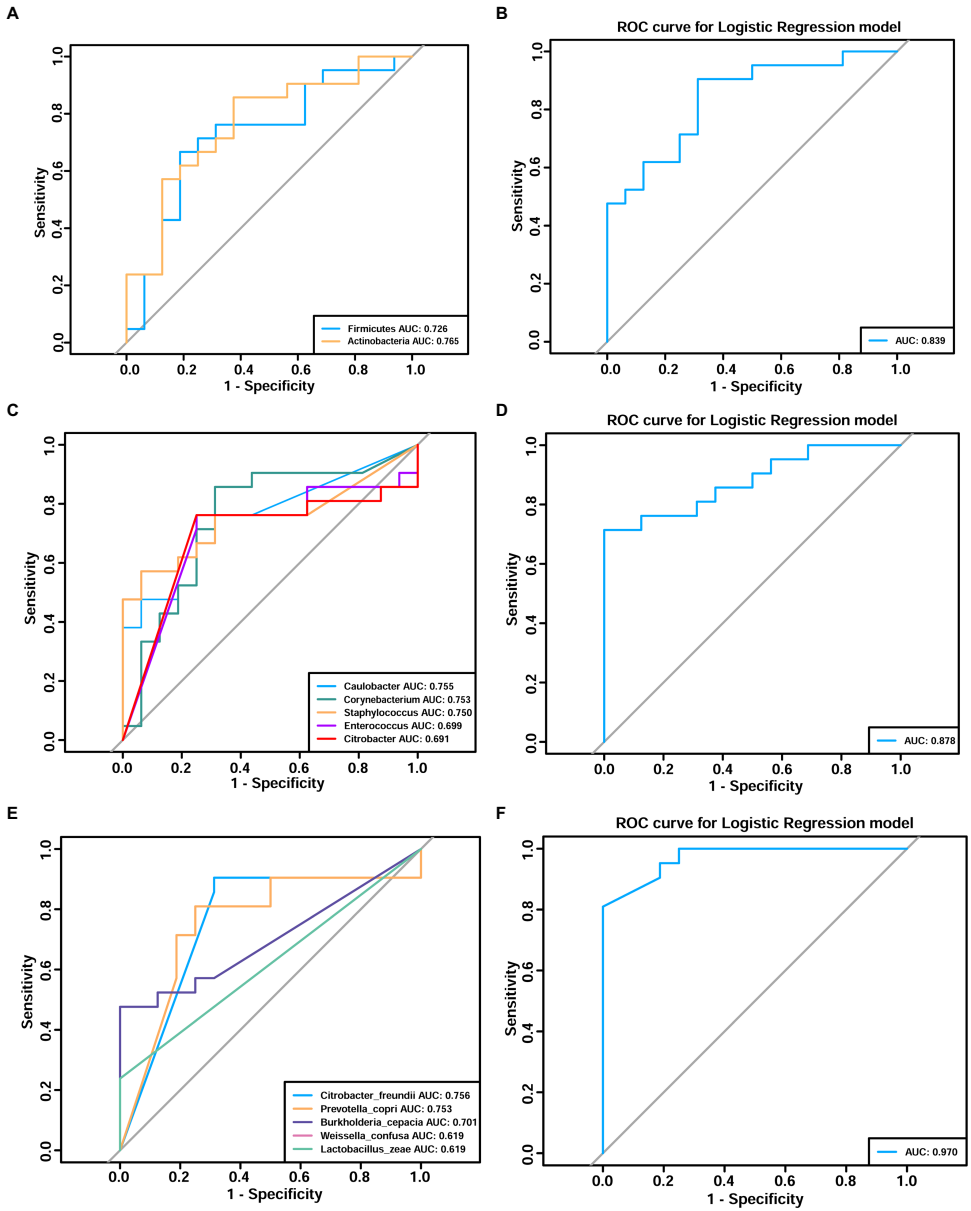
Receiver operating characteristic (ROC) analyses demonstrating the adenomyosis diagnosis efficacy of the endometrial microbiome at the phylum **(A,B)**, genus **(C,D)**, and species **(E,F)** levels. Different colors correspond to different taxa. Area under the curve (AUC) analysis was used to quantify the accuracy of adenomyosis diagnosis (an AUC value >0.7 was acceptable).

Next, receiver operating characteristic (ROC) curves were created to identify potential microbiome biomarkers at the phylum, genus, and species level for distinguishing patients with adenomyosis from those without adenomyosis (Figure 7). Area under the curve (AUC) analysis was then used to quantify the accuracy of adenomyosis diagnosis; an AUC > 0.7 was acceptable. At the phylum level, the AUC values for *Firmicutes* and *Actinobacteria* for the diagnosis of adenomyosis were 0.726 and 0.765, respectively (Figure 7A). At the genus level, Figure 7C depicts five curves representing *Caulobacter, Corynebacterium*, *Staphylococcus*, *Enterococcus,* and *Citrobacter*, respectively; the AUC values were 0.755, 0.753, 0.750, 0.699, and 0.691, respectively. At the species level, ROC curve analyses indicated the following diagnostic accuracies: *Citrobacter freundii* (AUC = 0.756), *Prevotella copri* (AUC = 0.753), and *Burkholderia cepacia* (AUC = 0.701) (Figure 7E). Notably, the integration of significantly different bacteria into logistic regression models indicated significantly better AUC values at the phylum (AUC = 0.839, sensitivity = 90.5%, specificity = 68.80%), genus (AUC = 0.878, sensitivity = 71.4%, specificity = 100%), and species levels (AUC = 0.970, sensitivity = 81%, specificity = 100%) (Figures 7B,D,F). These results indicate that *Citrobacter freundii*, *Burkholderia cepacia,* and *Prevotella copri* might serve as predictive markers for adenomyosis. Combined with biomarkers at the genus level, the diagnostic sensitivity and specificity for adenomyosis were 71.4 and 100.0%, respectively. However, the diagnostic sensitivity of adenomyosis at the species level increased to 81%.

### 3.7. Predictive functional profiling of endometrial microbiota

**Figure 8:**
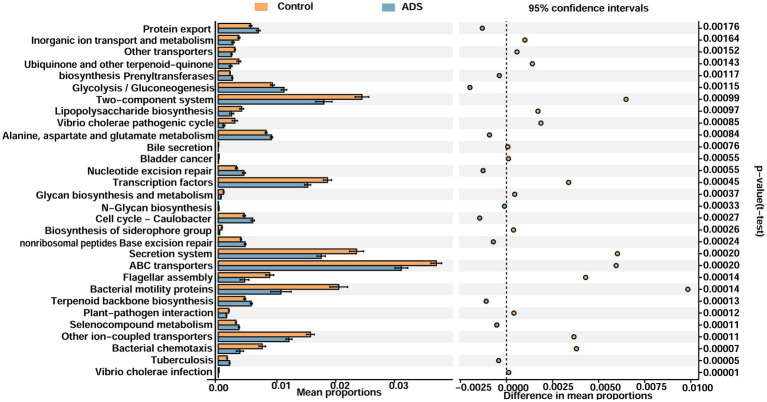
Functional analyses of the endometrial microbiota of participants in the two groups (level 3). Microbiota differences between groups as shown by differentially functional pathways. Downregulated pathways imply a lower ratio of the mean proportion of expression. Upregulated pathways suggest a higher ratio of the mean proportion of expression. White’s non-parametric *t*-test was used to calculate *p-*values.

PICRUSt was performed to explore the potential functional profiling of endometrial microbiota from the two groups. Supplementary Figure S2 illustrates several dysfunctional pathways among the two groups. On level 1, five functions identified as being statistically significant (*p* < 0.05): metabolism and genetics and genetic information processing were upregulated in the ADS group when compared with the control group (*p* = 0.0074 to *p* = 0.02446, mean proportion > 1%), while environmental information processing was upregulated in the control group when compared with the ADS group (*p* = 0.00168, mean proportion > 1%) (Supplementary Figure S3). On level 2, 18 functions were shown to be statistically significant (*p* < 0.05); the ADS patients exhibited upregulation in several pathways, including nucleotide metabolism, replication and repair, translation, enzyme families, and carbohydrate metabolism (*p* = 0.00267 to *p* = 0.021, mean proportion > 1%). In contrast, the control group exhibited upregulation in other pathways, including metabolism, signal transduction, cellular processes and signaling, and cell motility (*p* < 0.00001 to *p* = 0.02658, mean proportion > 1%) (Supplementary Figure S4). On level 3, 30 functions were identified as being statistically significant (*p* < 0.05). Compared with the control group, the ADS group exhibited upregulation in several pathways, including protein export, glycolysis/gluconeogenesis, alanine, aspartate, and glutamate metabolism (*p* < 0.001 to *p* = 0.00176, mean proportion > 1%). Compared with the ADS group, the control group exhibited upregulation in certain other pathways, including the two-component system, transcription factors, the secretion system, ABC transporters, other ion-coupled transporters, and bacterial motility proteins (*p* = 0.00011 to *p* = 0.0016, mean proportion > 1%) (Figure 8).

## 4. Discussion

In this study, we compared the endometrial microbiota from patients with and without adenomyosis, as confirmed by pathological analysis. We demonstrated the presence of an endometrial microbiota in the uteri of patients with adenomyosis that had a significantly lower richness than the control group. Until now, we know little about the precise etiology involved in the reduced richness of the endometrial microbiota in patients with adenomyosis. There were no significant differences between the two groups of patients with regard to their age, BMI, menstrual cycle phase, parity, and methods of contraception. In addition, we excluded patients with autoimmune disorders and genital infections. In contrast to our findings (mean age > 45 years old), one previous study found that the richness of the endometrial flora in patients with endometriosis (mean age < 36 years old) was increased (Wessels et al., 2021); this finding might be due to differences in participant age. Wang et al. (2021) reported that the richness of the uterine microbiome in the 40–50-year age group was lower than that in younger women. To a certain extent, such changes in the diversity of the endometrial microbiota may be the result of changes in the endometrial immune microenvironment, including endometrial immune response and inflammation (abnormal activation and differentiation of dendritic cells, macrophages, and Natural killer cells) which ultimately reduce endometrial receptivity, disrupt embryo implantation, and promote the progression of disease (Wira et al., 2005; Chen et al., 2021).

Furthermore, we found that the majority of samples could be clustered according to disease status by performing beta diversity analysis; this indicated that the structure and composition of the endometrial microbiota of patients with or without adenomyosis were different. LEfSe analysis also identified some biomarkers that were strictly associated with adenomyosis that might be useful for the diagnosis of adenomyosis. A high abundance of *Firmicutes* and *Actinomycetes* was detected in endometrial samples from patients with adenomyosis, whereas *Proteobacteria* and *Thermotogae* were present in high abundance in patients from the control group. These results are consistent with previous studies of endometrial cancer, intrauterine adhesions, endometrial polyps, and patients with infertility (Fang et al., 2016; Benner et al., 2018; Walsh et al., 2019; Qiu et al., 2021). In a previous study, Khan et al. studied patients with endometriotic cysts and observed a higher proportion of *Corynebacterium* (*Actinomycetes*, Gram-positive), *Enterococcus* (*Firmicutes*, Gram-positive), *Staphylococcus* (*Firmicutes*, Gram-positive), *Caulobacter* (*Proteobacteria*, Gram-negative), and *Citrobacter* (*Proteobacteria*, Gram-negative) in patients with adenomyosis (Akiyama et al., 2019). However, Chen et al. (2017) reported that *Methylophilaceae* (*Proteobacteria*, Gram-negative) was a useful biomarker for the endometrial flora in patients with adenomyosis. In the present study, blank and negative controls were used to eliminate potential bacterial contamination from sampling collection, DNA processing, library preparation, and from the local environment. Differences between our results and those published previously may be due to analytical differences, along with different collection methods and study populations. Moreover, our results align with the bacterial contamination hypothesis of endometriosis that Gram-negative bacteria show enrichment in the endometrium of patients with endometriosis. Lipopolysaccharide (LPS) is a component of the outer membrane of Gram-negative bacteria and regulates the uncontrolled activation of inflammatory and immune responses in the pelvis and the growth of endometriosis *via* the LPS/TLR4 cascade (Khan et al., 2018). The major taxa of relative richness (TOP 10) represented in the population of patients with adenomyosis were non-*Lactobacillus* dominant (*Lactobacillus* species <90%, NLD), including *Lactobacillus*, *Corynebacterium*, *Streptococcus*, *Prevotella*, *Hyphomicrobium*, *Geobacillus* (3.78%), and *Vibrio*. These results are consistent with those reported previously by Chen et al. (2017) but differed from several other studies which reported that the most dominant endometrial microbiota are *Lactobacillus* (*Lactobacillus* species >90%, LD) (Moreno et al., 2016; Tao et al., 2017; Kyono et al., 2018).

Although the precise mechanisms underlying the endometrial microbiota with regard to human health and disease remain unclear, an imbalance of the microbiota is closely related to the pathophysiological mechanisms of endometriosis, endometrial cancer, and uterine fibroids. Currently, most of our knowledge relating to the interaction between bacteria and their host is derived from the highly diverse intestinal microbiota (Belkaid and Hand, 2014). The diversity of the endometrial microbiota is lower than that of the intestinal microbiota and higher than that of the vaginal microbiota (Chen et al., 2017). At the species level, our results indicated that *Citrobacter freundii* (Gram-negative), *Burkholderia cepacia* (Gram-negative), and *Prevotella copri* (Gram-negative) could be biomarkers for adenomyosis. *Citrobacter freundii* is a symbiotic bacterium found in the intestines of humans and animals (Liu et al., 2015). However, another study reported that *Citrobacter freundii* activated inflammation *via* the nucleotide oligomeric Domain-Like receptor family, a pyridine domain containing 3 (NLRP3) inflammatory body, in a T6SS-dependent manner (Liu et al., 2021). *Citrobacter freundii* can also invade, reproduce, and transfect human microvascular endothelial cells *in vitro* (Badger et al., 1999); this was associated with neonatal meningitis and brain abscesses (Joaquin et al., 1991). *Burkholderia cepacia* was previously shown to secrete BceF Tyrosine Kinase which participates in the formation biofilm and contributes to respiratory tract infection. BceF tyrosine kinase does not show any homology with human kinase and is therefore useful for the development of target antibacterial drugs (Mayer et al., 2021). Another study revealed that ‘quorum sensing’ (QS) enabled *Burkholderia cepacia* to produce virulent factors and biofilms, thus destroying the host’s innate immunity, especially epithelial cells, neutrophils, and megaphagocytes in patients with cystic fibrosis (Ganesh et al., 2020). Similar results were observed in patients with keratitis (Ho et al., 2021). Wessels et al. also reported that *Burkholderia* enriched the endometrium of symptomatic patients with pelvic pain but without endometriosis, as confirmed by surgery (Wessels et al., 2021).

In addition, *Prevotella copri*, a potential pro-inflammatory bacterium, has been associated with inflammation and autoimmunity. In a mouse model of glucan sulfate-induced colitis, elevated levels of *Prevotella copri* richness were associated with more severe intestinal inflammation than mice without *Prevotella copri* (Cao et al., 2020). *Prevotella copri* was also positively correlated with the severity of rheumatoid arthritis (Scher et al., 2013). Recent studies have revealed that *Prevotella copri* play an important role in anti-tumor therapy; patients with a high abundance of *Prevotella copri* could benefit from treatment (Jin et al., 2019). In addition, Yu et al. (2019) proved that *Prevotella copri* caused damage to the mucosal barrier by expressing reduced levels of tight junction proteins (ZO-1, Occludin, and Claudin-1) and increased expression levels of inflammatory factors (IFN-γ, IL-1 β, IL-6, and MCP-1). The eutopic endometrium is thought to play a vital role in the pathogenesis of adenomyosis. Studies have predicted that the IL-6 and ERK/MAPK pathways are abnormally activated in the eutopic endometrium of patients with adenomyosis (Xiang et al., 2019). Furthermore, the numbers of natural killer cells and macrophages have been found to be elevated in the eutopic endometrium of patients with adenomyosis along with increased levels of inflammatory markers (IL-1b and IL-6) (Zhihong et al., 2016; Carrarelli et al., 2017).

Although the role of the microbiota has been revealed in some diseases, we explored the potential mechanisms that may act between the host and microbes by applying the PICRUSt algorithm. Differentially regulated pathways between the two groups that may be of relevance included genetic information processing and metabolism; these were both enriched in the ADS group when compared to the control group. Furthermore, nucleotide metabolism, replication and repair, translation, enzyme families, and carbohydrate metabolism pathways were all upregulated in the ADS group when compared to the control group. Furthermore, protein export, glycolysis/gluconeogenesis, alanine aspartate, and glutamate metabolism were all enriched in the ADS group when compared to the control group. Only limited research has targeted the endometrial microbiota of women with adenomyosis. *Citrobacter freundii*, *Burkholderia cepacia*, and *Prevotella copri* are pathogenic microbiota that may be associated with adenomyosis; these are Gram-negative bacteria that secrete LPS *via* protein export, which causes mucosal inflammation and damages the mucosal barrier integrity (Mobley et al., 2001). Microorganisms and their metabolites can also trigger DNA damage and mutation of the epithelial cells and directly regulate the biological function of host cells *via* increased proliferation or reduced apoptosis (Lim et al., 2017; Chattopadhyay et al., 2019; Helmink et al., 2019).

Our study has several strengths that should be considered. We used a blinded approach to ensure the accuracy in all statistical analyses. We also took multiple methods to minimize the contamination. Furthermore, all patients in this study received pathological examination to guarantee the precision and accuracy of diagnosis. However, this study has several limitations that should be considered. First, the patients in this study were ethnically and genetically homogenous. Second, the endometrial microbiota was susceptible to contamination as a specimen with low microbial abundance. Researchers tried to avoid contamination at all times during sampling; however, environmental contamination cannot be ruled out. Third, all participants received hysterectomy in this study; therefore, we were unable to recruit healthy samples for the control group; this would have provided a better understanding of the differences in endometrial microbiota between the patients with adenomyosis and healthy women. Finally, another limitation of this study is its small sample size; however, other studies on similar topics also involved small sample sizes. In the future, studies should aim to recruit a larger population size for investigation.

## 5. Conclusion

In conclusion, females possess an endometrial microbiota that contains various microbes. Adenomyosis is highly associated with endometrial bacterial dysbiosis. A potential variation in the microbial environment of the endometrium might contribute to adenomyosis. Pathogenic bacteria, such as *Citrobacter freundii*, *Burkholderia cepacia*, and *Prevotella copri*, could be considered as potential emerging causes of adenomyosis. Further studies should investigate the cross-talk between potential pathogenic microbiota in patients with adenomyosis, evaluate whether the early diagnosis of adenomyosis could be assessed according to the endometrial microbiota profile, and investigate whether the endometrial microbiota could be modified to increase treatment outcomes.

## Data availability statement

The data presented in the study are deposited in the Sequence Read Archive repository (https://www.ncbi.nlm.nih.gov/sra/), accession number PRJNA891223.

## Ethics statement

The studies involving human participants were reviewed and approved by Medical Ethics Committee of Beijing Obstetrics and Gynecology Hospital, Capital Medical University No. 2016-KY-012-02. The patients/participants provided their written informed consent to participate in this study.

## Author contributions

QL, HD, ShW, ZG, and YC conceived the study and reviewed and edited the manuscript. QL, SiW, CC, MS, HS, and CZ participated in sample collection. QL and ZG conducted the data analysis and prepared the figures and tables. QL helped to write the original draft. All authors contributed to the article and approved the submitted version.

## Funding

This research was funded by the National Natural Science Foundation of China (grant number 81571412) and supported by the Beijing Obstetrics and Gynecology Hospital, Capital Medical University (grant number FCYY201920).

## Conflict of interest

The authors declare that the research was conducted in the absence of any commercial or financial relationships that could be construed as a potential conflict of interest.

## Publisher’s note

All claims expressed in this article are solely those of the authors and do not necessarily represent those of their affiliated organizations, or those of the publisher, the editors and the reviewers. Any product that may be evaluated in this article, or claim that may be made by its manufacturer, is not guaranteed or endorsed by the publisher.

## Footnotes

^1^ http://greengenes.lbl.gov

^2^ http://www.ehbio.com/ImageGP/index.php/Home/Index/LEFSe.html

^3^ https://github.com/picrust/picrust2/wiki

^4^ http://www.genome.jp/kegg/
