# Supplementary material for: Endometrial microbiota in women with and without adenomyosis: A pilot study
Source: Front Microbiol. 2023 Jan 20;14:1075900. doi: 10.3389/fmicb.2023.1075900 (PMC9895119; doi:10.3389/fmicb.2023.1075900)
Supplement: Supplementary file 2 [file Data_Sheet_1.docx]

Supplementary Material

# Supplementary Table S1

**Supplementary Table S1**. Number of raw reads, clean reads and the Q30% for all samples

| Samples | | raw reads | clean reads | Q30% |
| --- | --- | --- | --- | --- |
| Control_01 | 101305 | 98479 | 92.77 |  |
| Control_02 | 102425 | 89198 | 92.76 |  |
| Control_03 | 105182 | 104521 | 92.02 |  |
| Control_04 | 85402 | 70342 | 89.18 |  |
| Control_05 | 87296 | 78117 | 90.32 |  |
| Control_06 | 86269 | 62948 | 89.07 |  |
| Control_07 | 102435 | 99027 | 92.12 |  |
| Control_08 | 108678 | 98666 | 92.64 |  |
| Control_09 | 100701 | 97796 | 92.45 |  |
| Control_10 | 100642 | 99657 | 92.34 |  |
| Control_11 | 101046 | 99869 | 92.75 |  |
| Control_12 | 105562 | 100640 | 92.46 |  |
| Control_13 | 103855 | 94942 | 92.45 |  |
| Control_14 | 106639 | 104948 | 92.04 |  |
| Control_15 | 109408 | 100931 | 92.46 |  |
| Control_16 | 108517 | 105484 | 91.60 |  |
| Control_17 | 102351 | 101915 | 91.90 |  |
| ADS_01 | 101861 | 98150 | 93.07 |  |
| ADS_02 | 103339 | 91543 | 92.85 |  |
| ADS_03 | 104986 | 103501 | 92.93 |  |
| ADS_04 | 101743 | 101557 | 93.50 |  |
| ADS_05 | 108188 | 107491 | 92.91 |  |
| ADS_06 | 105232 | 104670 | 93.41 |  |
| ADS_07 | 82242 | 65702 | 89.29 |  |
| ADS_08 | 86205 | 68994 | 89.98 |  |
| ADS_09 | 80345 | 66277 | 89.48 |  |
| ADS_10 | 83614 | 80684 | 89.42 |  |
| ADS_11 | 84338 | 34121 | 89.51 |  |
| ADS_12 | 84005 | 48690 | 87.68 |  |
| ADS_13 | 80986 | 62983 | 89.16 |  |
| ADS_14 | 84152 | 43140 | 86.81 |  |
| ADS_15 | 81676 | 49126 | 86.45 |  |
| ADS_16 | 84850 | 63750 | 87.87 |  |
| ADS_17 | 105373 | 89005 | 87.87 |  |
| ADS_18 | 104251 | 101640 | 92.44 |  |
| ADS_19 | 109850 | 95203 | 91.35 |  |
| ADS_20 | 107482 | 97333 | 92.17 |  |
| ADS_21 | 101394 | 96840 | 92.08 |  |
| Total | 3703825 | 3277880 | / |  |
| Average | 97469.07895 | 86260 | 91.12 |  |

# Supplementary Figures

## Supplementary Figure S1


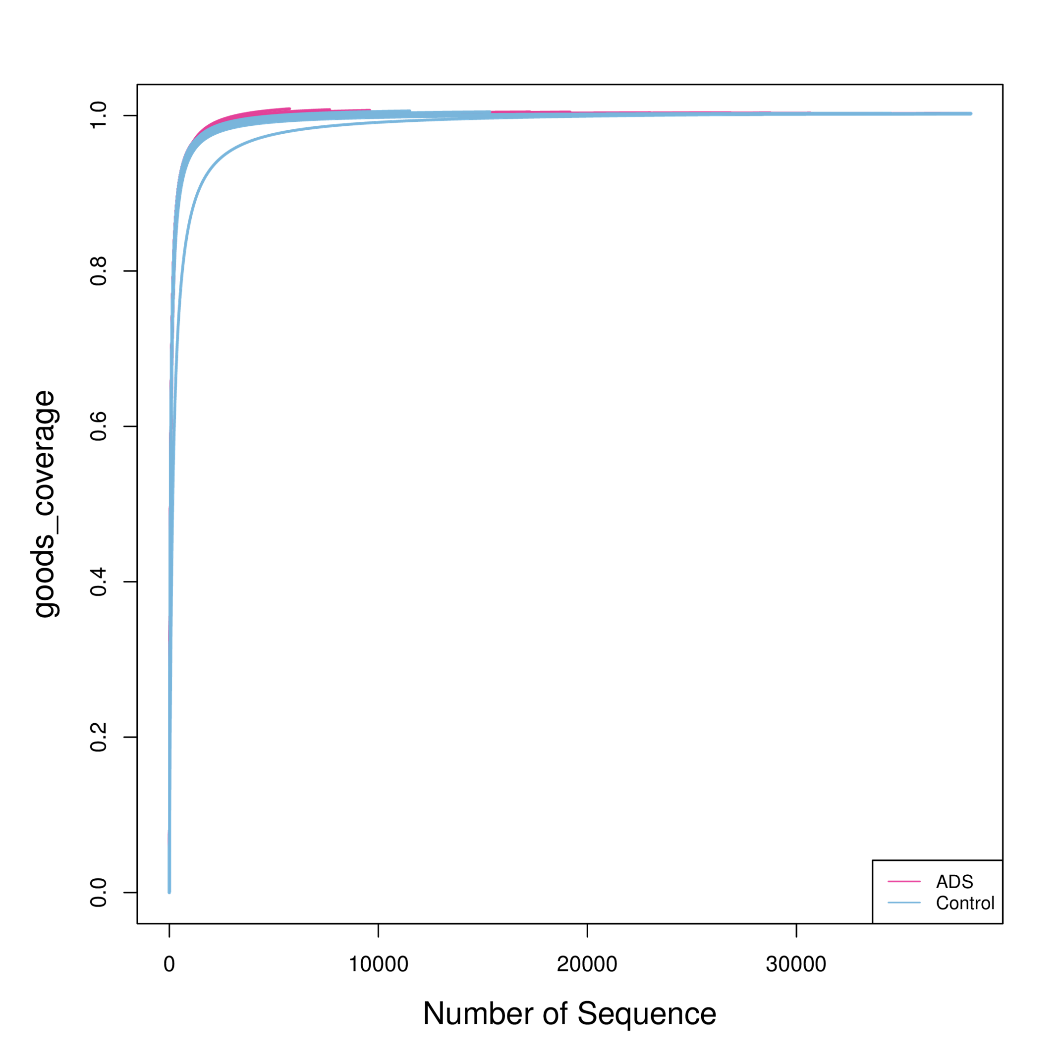


**Supplementary Figure S1.** Alpha rarefaction was used to assess the depth of sequencing in the two groups. The red line represents the adenomyosis (ADS) group and the blue line represents the control group.

## Supplementary Figure S2


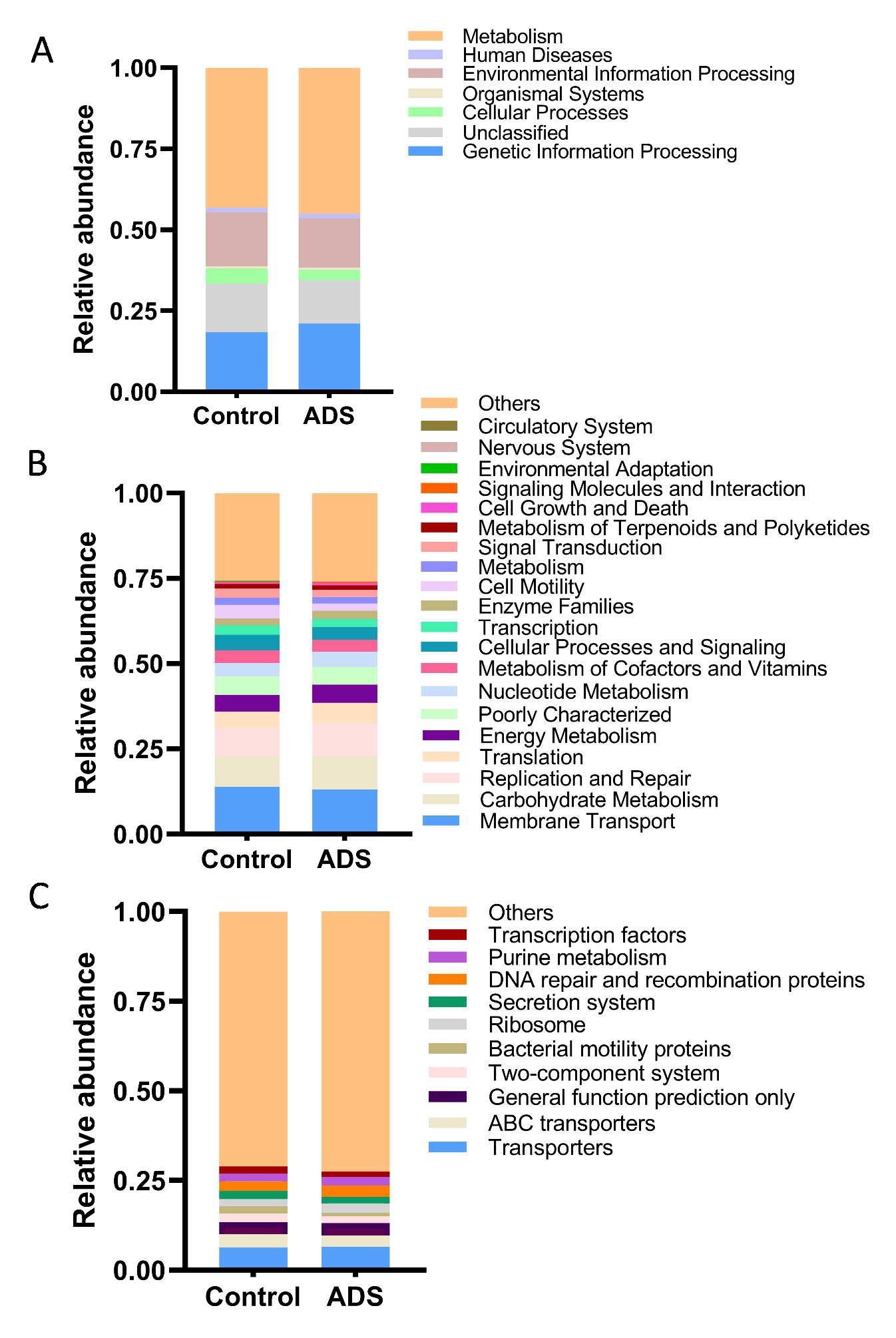


**Supplementary Figure S2.** The proportion of predictive functional profiling of the endometrial microbiota in the two groups. The relative proportion of gene function prediction on level 1 (A), level 2 (B) and level 3 (C) in each group is shown in column charts.

## Supplementary Figure S3


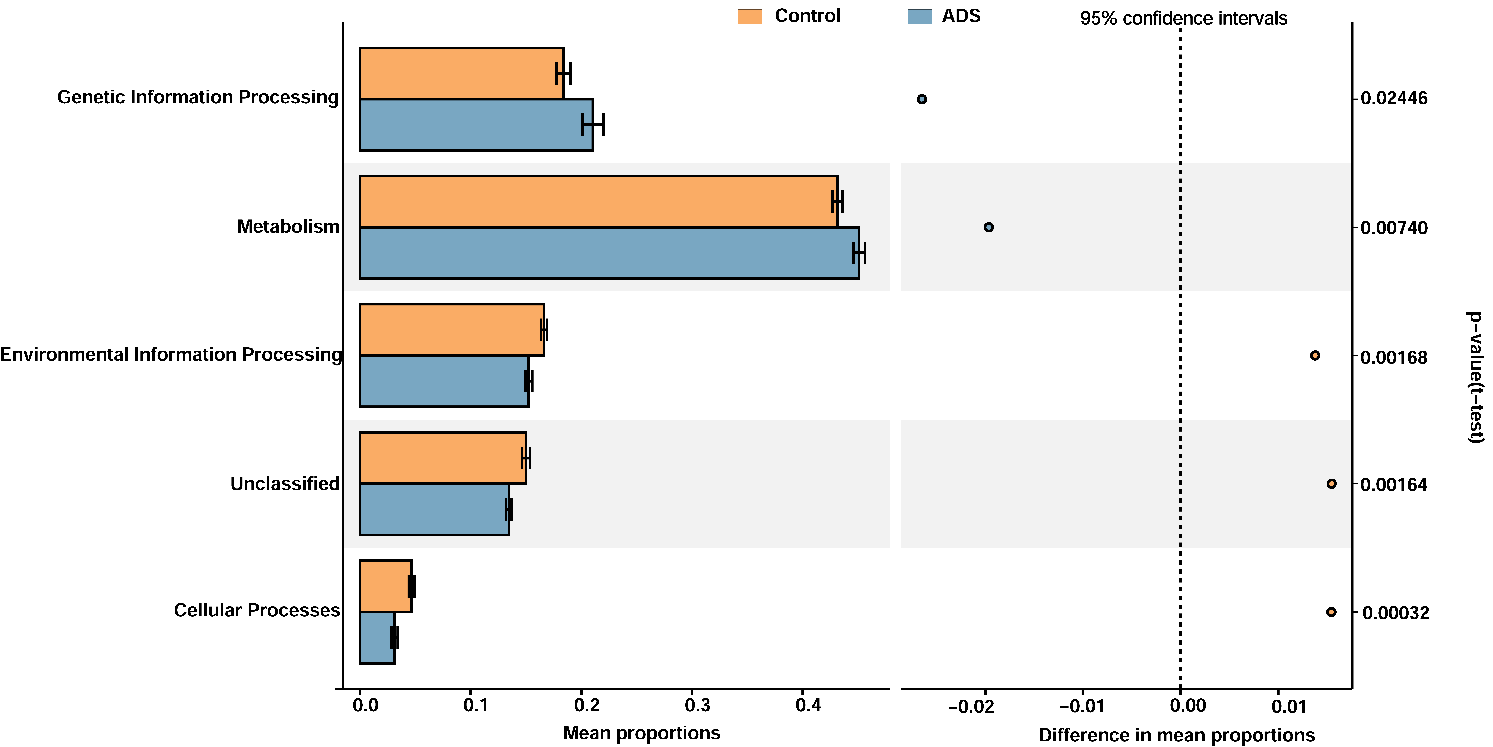


**Supplementary Figure S3.** Functional analyses of the endometrial microbiota of participants in the two groups (level 1). Microbiota differences between groups as shown by differentially functional pathways. Downregulated pathways imply a lower ratio of the mean proportion of expression. Upregulated pathways indicate a higher ratio of the mean proportion of expression. White’s non-parametric t-test was used to calculate *p* values.

## Supplementary Figure S4


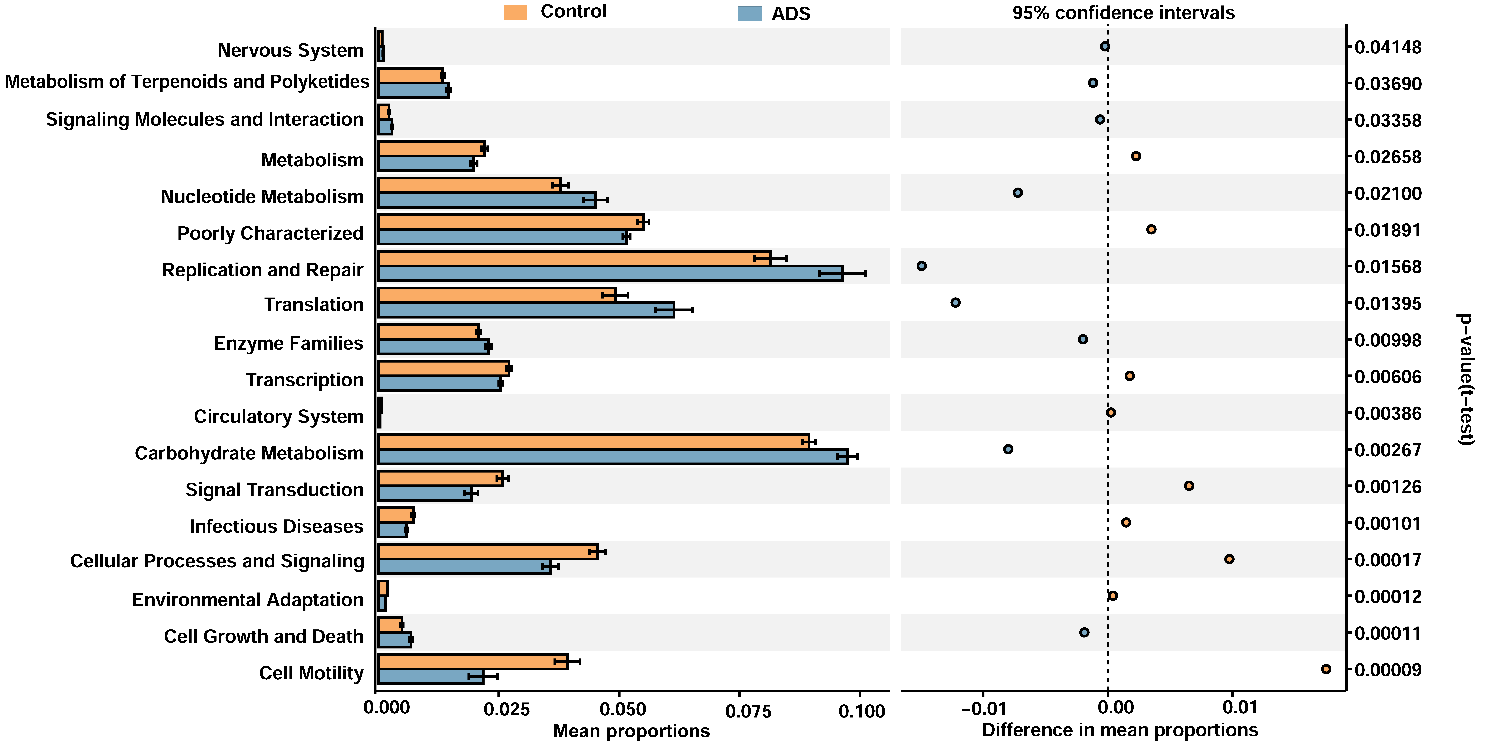


**Supplementary Figure S4.** Functional analyses of the endometrial microbiota of participants in the two groups (level 2). Microbiota differences between groups as shown by differentially functional pathways. Downregulated pathways imply a lower ratio of the mean proportion of expression. Upregulated pathways imply a higher ratio of the mean proportion of expression. White’s non-parametric t-test was used to calculate *p* values.
